# Identification of robust reference genes for studies of gene expression in FFPE melanoma samples and melanoma cell lines

**DOI:** 10.1097/CMR.0000000000000644

**Published:** 2019-09-24

**Authors:** Julie N. Christensen, Henrik Schmidt, Torben Steiniche, Mette Madsen

**Affiliations:** a Department of Biomedicine, Aarhus University; Departments of b Oncology; c Pathology, Aarhus University Hospital, Aarhus N, Denmark

**Keywords:** biomarker, cell lines, formalin-fixed paraffin-embedded, gene expression, melanoma, normalization, qRT-PCR, reference genes

## Abstract

There is an urgent need for novel diagnostic melanoma biomarkers that can predict increased risk of metastasis at an early stage. Relative quantification of gene expression is the preferred method for quantitative validation of potential biomarkers. However, this approach relies on robust tissue-specific reference genes. In the melanoma field, this has been an obstacle due to lack of validated reference genes. Accordingly, we aimed to identify robust reference genes for normalization of gene expression in melanoma. The robustness of 24 candidate reference genes was evaluated across 80 formalin-fixed paraffin-embedded melanomas of different thickness, −/+ ulceration, −/+ reported cases of metastases and of different BRAF mutation status using quantitative real-time PCR. The expression of the same genes and their robustness as normalizers was furthermore evaluated across a number of melanoma cell lines. We show that housekeeping genes like *GAPDH* do not qualify as stand-alone normalizers of genes expression in melanoma. Instead, we have as the first identified a panel of robust reference genes for normalization of gene expression in melanoma tumors and cultured melanoma cells. We recommend using a geometric mean of the expression of *CLTA, MRPL19* and *ACTB* for normalization of gene expression in melanomas and a geometric mean of the expression of *CASC3* and *RPS2* for normalization of gene expression in melanoma cell lines. Normalization, according to our recommendation will allow for quantitative validation of potential novel melanoma biomarkers by quantitative real-time PCR.

## Abstract 2

Supplemental Digital Content is available in the text.

## Introduction

Due to the aggressiveness and lethality of metastatic melanoma cancer [1], many research groups have attempted to identify and characterize novel melanoma biomarkers that can predict increased risk of metastasis at an early stage. Established melanoma biomarkers like Breslow thickness and ulceration unfortunately fail to detect all melanomas in risk of metastatic spread [2,3] resulting in metastatic relapse at later time points. Accordingly, novel and better melanoma markers are wanted.

Nowadays screening for novel biomarkers is frequently performed using advanced technologies like Next Generation Sequencing (NGS) or RNA sequencing (RNAseq). However, simpler PCR-based methods, like quantitative real-time PCR (qRT-PCR), are routinely chosen for validation of potential biomarkers identified by these more advanced technologies [4]. The rationale being the cost-benefit of the analysis and that PCR-based methods do not require bioinformatics contrary to NGS or RNAseq data analysis.

For gene expression analyses and relative quantification (RQ), qRT-PCR remains one of the most applied technologies. A consensus on good qRT-PCR practice has been established through extensive experience with the technique [5]. Reliable RQ of gene expression levels relies on suitable reference genes for normalization [6,7] and comparable RNA integrity between the samples under investigation [5,8].

The robustness of qRT-PCR reference genes did not receive much attention in the early days of qRT-PCR and ‘housekeeping’ genes like *GAPDH* were continuously used for normalization of gene expression despite lack of evidence of their the robustness [9,10]. Actually, more and more studies have established that ‘housekeeping’ genes are not always stably expressed across a cohort [6,11,12]. Follow-up studies have shown that false conclusions may have been made because unstable reference genes were applied for normalization of target gene expression levels [11,13,14]. Nevertheless, studies are still published today where the stability and robustness of the reference genes used for normalization of target gene expression has not been properly validated, and traditional housekeeping genes are still applied as normalizers, despite the risk of introducing bias [15–17].

The vast majority of all biomarker studies are unsuccessful, often due to failure in reproduction of study results and in the ability to reach statistical significance [18–22]. A critical obstruction might be the lack of properly validated reference genes for normalization of target gene expression measured in the samples under investigation.

Aiming to pave the way for successful melanoma biomarker studies and for optimization of qRT-PCR-based analyses of gene expression in melanoma samples in general, we set out to identify and validate robust melanoma reference genes.

## Methods

### FFPE melanoma samples and melanoma cell lines

Melanoma FFPE tissue specimens were selected from the archives at the Department of Pathology, Aarhus University Hospital, Aarhus, Denmark. FFPE melanoma blocks established upon surgical resection of primary melanomas from 93 patients were included in this study. Of these 93 samples, 13 were used for the initial study of *ACTB* and *GAPDH* stability, whereas the other 80 samples were used for the main study of the stability of 24 selected reference gene candidates. From each melanoma block 3–6 sections of 10 µM were sliced. All sections were macrodissected before RNA purification; the skin samples based on E-cadherin stainings of parallel sections and the melanoma samples based on E-cadherin and Melan A stainings of parallel sections. The study was approved by the local Ethical Committee (journal numbers; 48648 and 55836).

The human melanoma cell lines FM3 [European Searchable tumor line database (ESTDAB)-007], FM82 (ESTDAB-027), FM88 (ESTDAB-029) and FM92 (ESTDAB-032) were used in this study and were a kind gift from Professor Per Guldberg at The Danish Cancer Society [23,24]. Sub-clonal cell lines of the parental FM3 and FM88 cell lines were previously established [25], and the following cell lines were used in this study; 31-D3, 31-D8, 35-E1, 35-F1, 35-G7 of FM3 and 881-D9, 881-H6, 885-D3, 885-D10 of FM88. Cells were grown in RPMI 1640 (Life Technologies, Naerum, Denmark) supplemented with 10% heat-inactivated fetal bovine serum (BioWest, VWR, Herlev, Denmark), 100 U/ml penicillin and 100 µg/ml streptomycin, and 2 mM L-glutamine (Life Technologies) under normoxic condition at 37°C. Passaging was performed at 70%–90% confluence, and cells were used in low passages. Cells were pelleted on ice in 140 mM NaCl, 10 mM HEPES [4-(2-hydroxyethyl)-1-piperazineethanesulfonic acid], 2 mM CaCl_2_, 1 mM MgCl_2_, pH 7.8 by centrifugation at 2000 rpm for 1 minute and stored at −80°C until RNA purification.

### RNA extraction and cDNA synthesis

RNA was extracted from cell culture pellets and sections of paraffin-embedded tissue samples using the RNeasy Mini Kit and the RNeasy FFPE Kit (both Qiagen, Copenhagen, Denmark), respectively. An additional on-column DNase (Qiagen) digestion step was performed to remove genomic DNA. mRNA was converted into cDNA using the High-Capacity cDNA Reverse Transcription Kit on a Verity Thermal Cycler (both Applied Biosystems, Naerum, Denmark). gDNA background was evaluated in no reverse transcriptase control reactions.

### RNA quality test by multiplex *GAPDH* Reverse Transcription-PCR

A multiplex reverse transcription-PCR (RT-PCR) was performed using the OneStep RT-PCR kit (Qiagen) on a Verity Thermal Cycler (Applied Biosystems) with the following *GAPDH* forward (FW) and reverse (R) primers in combination; hGAPDH_FW: 5′-CGA CAG TCA GCC GCA TCT T-3′ (h for human), hGAPDH_R1: 5′-CCC CAT GGT GTC TGA GCG-3′, product size with FW-primer: 62 bp, hGAPDH_R2: 5′-AAG CAG CCC TGG TGA CCA G-3′, product size with FW-primer: 123 bp, hGAPDH_R3: 5′-GCC ATG GAA TTT GCC ATG GG-3′, product size with FW-primer: 230 bp, hGAPDH_R4: 5′-CCA GCA TCG CCC CAC TTG A-3′, product size with FW-primer: 328 bp. This cocktail of primer pairs for amplification of *GAPDH* RT-PCR products of varying lengths was used to evaluate the quality of RNA extracted from sections of FFPE blocks. SYBR safe DNA gel stain (Thermo Fisher Scientific, Hvidovre, Denmark) and Gel loading dye (BioNordika, Herlev, Denmark) was used to visualize RT-PCR products using a Fuji LAS-3000 Imaging System (Fujifilm Europe GmBH, Düsseldorf, Germany) with IMAGE READER LAS-3000 software version 2.2 (Science Imaging Scandinavia AB, Saltsjö-Boo, Sweden).

### RNA integrity measurements

RNA integrity was measured for RNA extracted from the 80 FFPE melanoma samples of the main study cohort and from the 13 cell lines using the RNA 6000 Nano Kit and a 2100 Bioanalyzer (Agilent Technologies, Glostrup, Denmark) according to manufacturer’s instructions. Results were evaluated using the 2100 Expert software (Agilent Technologies). RNA Integrity Numbers (RIN values) are displayed in Table of Supplemental Digital Content 1, http://links.lww.com/MR/A190.

### Selection of reference gene panel

Candidate reference genes for gene expression analysis were selected from papers available in the Pubmed database and extracted using the following keyword combinations: “PCR” AND “reference” AND “melanoma”, “genes”[Mesh] AND “melanoma”[Mesh] AND “reference”, “qPCR” AND “melanoma”[Mesh], and from traditionally used reference genes such as *ACTB*, *GAPDH* and *B2M*. In addition, reference genes suggested using the RefGene Tool (GENEVESTIGATOR, NEBION/ETH Zurich, 2008) [26] based on the following sample selections/datasets; *Neoplasms malignant melanoma of skin*, *Oncology Skin neoplasm melanoma-HS_AFFY_U133A and Oncology Skin neoplasm melanoma-HS_AFFY_U133PLUS_2* were included [27–34]. Further filtering of candidate reference genes was performed to ensure a minimum of genes with overlapping functions or from similar or associated pathways. Furthermore, genes were included only if high-quality TaqMan assays were available. The 24 selected genes are shown in Table 1.

**Table 1:**
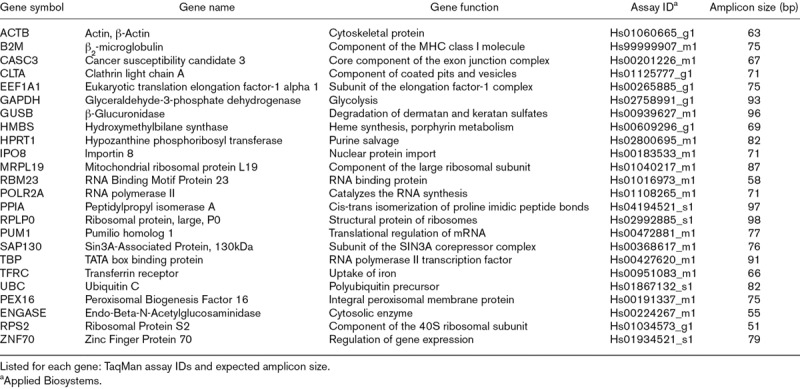
Overview of candidate reference genes included in the study

### qRT-PCR

Gene-specific TaqMan assays were purchased from Applied Biosystems. All assays were designed to give amplicons of less than 100 base pairs (bp). Assay IDs are included in Table 1. The following TaqMan assays were applied: *Candidate reference genes: ACTB:* Hs01060665_g1, *B2M:* Hs99999907_m1, *CASC3:* Hs00201226_m1, *CLTA:* Hs01125777_g1, *EEF1A1:* Hs00265885_g1, *GAPDH:* Hs02758991_g1, *GUSB:* Hs00939627_m1, *HMBS:* Hs00609296_g1, *HPRT1:* Hs02800695_m1, *IPO8:* Hs00183533_m1, *MRPL19:* Hs01040217_m1, *RBM23:* Hs01016973_m1, *POLR2A:* Hs01108265_m1, *PPIA:* Hs04194521_s1, *RPLP0:* Hs02992885_s1, *PUM1:* Hs00472881_m1, *SAP130:* Hs00368617_m1, *TBP:* Hs00427620_m1, *TFRC:* Hs00951083_m1, *UBC:* Hs01867132_s1, *PEX16:* Hs00191337_m1, *ENGASE:* Hs00224267_m1, *RPS2:* Hs01034573_g1, *ZNF70:* Hs01934521_s1. Target genes for reference gene validation experiment: *ACTB:* Hs01060665_g1, *GAPDH:* Hs02758991_g1, *LRP1:* Hs00233856_m1. All of the assays contained FAM-coupled probes. qRT-PCR was performed using Taqman Fast Advanced Master Mix (Applied Biosystems), and a Quantstudio 3 Fast Real-Time PCR cycler (Applied Biosystems) according to standard procedures. Contamination was evaluated by including no template control (NTC) reactions. Interplate variation was assessed by running an interplate control on a standard template for a study-unrelated target gene. All reactions were performed as technical triplicates. PCR efficiencies were calculated based on standard curves by the equation: PCR efficiency = (10^−1/slope^ − 1) × 100. PCR reaction efficiencies for the recommended assays were between 99.53% and 106.65%.

### Droplet digital PCR

Amplitude-based amplicon multiplexing droplet digital-PCR (ddPCR) approach was applied, allowing determination of the expression of three genes in one reaction; two reference genes and one target gene. Gene-specific TaqMan assays were purchased from Applied Biosystems; reference genes: *CASC3:* Hs00201226_m1 (FAM) and *RPS2:* Hs01034573_g1(VIC), target genes: *ACTB:* Hs01060665_g1(FAM), *GAPDH:* Hs02758991_g1(FAM), *LRP1:* Hs00233856_m1(FAM). ddPCR was performed by combining ddPCR Supermix for Probes (no dUTP), (BIO-RAD, Herlev, Denmark), TaqMan assays (Applied Biosystems) and a cDNA template. The procedure was performed on the QX200 Droplet Digital PCR System (BIO-RAD) with appropriate reagents and consumables (BIO-RAD). Standard settings were used for the end-point PCR reaction (40 cycles) as the absolute quantification mode for the droplet reader. Contamination was evaluated by including NTC reactions. ddPCR data were analyzed using QuantaSoft Analysis Pro Software (BIO-RAD). The absolute quantity of target genes was normalized using the geometric mean of *CASC3* and *RPS2* quantities, and relative expression ratios between the 13 cell lines were calculated from the normalized absolute quantities.

### RNA sequencing

Total RNA purified from two cell lines was sent for RNAseq at BGI Europe Genome Center (Copenhagen, Denmark). From total RNA samples, mRNA was purified using Oligo-(dT) magnetic beads. Purified mRNA was fragmented and reversely transcribed to cDNA using random hexamers and made double-stranded (ds). Adaptors were ligated to ends of ds cDNA, and ligation products were amplified by PCR using adaptor specific primers. Amplified PCR products were denatured and made cyclic by splint-oligos and DNA ligation. Before BGISEQ-500 library construction, RIN value, 28S/18S and the fragment length distribution and molar concentrations were analyzed using the RNA 6000 Nano Kit and a 2100 Bioanalyzer (Agilent Technologies) and evaluated in the 2100 Expert software (Agilent Technologies). Sequencing was performed on the BGISEQ-500 platform using single end sequencing strategy. Post sequencing low-quality reads, reads with adaptors and reads with unknown bases were filtered from the raw data to obtain clean data. Clean reads were mapped using HISAT algorithm to the Reference Genome Reference sequence and to the reference transcript using Bowtie2 software. RefSeq Assembly Accession matching GRCh38.p11 was applied as Reference Genome. Expression levels of genes were calculated by the FPKM method using the RSEM software package. FPKM data for the three selected target genes; *LRP1, ACTB* and *GAPDH*, were used for validation of *CASC3* and *RPS2* as reference genes by calculating the expression ratio for 31-D3 relative to 35-G7.

### Data analysis

Initial evaluation of qRT-PCR analysis data was performed using the QuantStudio Design & Analysis Software v 1.4.1 (Applied Biosystems). Data are reported as mean ± SD. qRT-PCR was performed as far as possible according to MIQE guidelines [5]. Gene expression stability was evaluated for all 24 candidates using the geNorm [35] and NormFinder [36] algorithms as Excel add-ins.

The geNorm algorithm is based on the estimation of gene expression ratios, defined as stability measures. For each potential reference gene, the pairwise variation (V) to all the other evaluated genes is estimated as the SD of Log_2_-transformed expression ratios. Finally, a gene stability measure (M) is estimated as the average pairwise variation for each gene. The M-value is thus used to evaluate the stability of the analyzed genes. The smaller the M-value, the more stably expressed is the reference gene. The determination of appropriate number of reference genes to include may be determined by evaluating the pairwise variation (V) on sequential addition to a normalization factor (NF).

The NormFinder algorithm is a model-based approach to estimating the variation in gene expression of candidate reference genes. The gene expression data is log-transformed and the intra- and inter-group variation estimated for the individual reference genes. The estimated intra- and inter-group variations are combined to form the NormFinder stability value, which directly describes the size of the systematic error added by each reference gene.

NormFinder has the advantage over geNorm in using subgroup estimates in the evaluation of reference gene stability. The final output for enabling calculation of the relative gene expression of target genes is a NF build from top-ranked genes estimated to introduce the least systematic error when applied as reference genes. The NF is calculated as the geometric average of the included reference genes. On an experimental basis, the number of reference genes to include is determined with regard to experimental design and by the acceptable level of variation. GraphPad Prism version 7 (GraphPad Software, Inc., La Jolla, California, USA) was used for graphic presentation of data and for correlation analysis of qRT-PCR and ddPCR data Pearson correlations were computed.

## Results

### Evaluation of the variation in *ACTB* and *GAPDH* expression levels amongst primary melanomas

The expression of *ACTB* and *GAPDH* in 13 primary melanomas was investigated by qRT-PCR. Multiple sections of each tumor were analyzed (minimum n = 3; maximum n = 6 per tumor), and inter- and intra-tumor variation in gene expression was evaluated to assess if *A*C*TB* and *GAPDH* could qualify as reference genes in qRT-PCR-based analysis of gene expression in melanoma tissue samples.

**Fig. 1:**
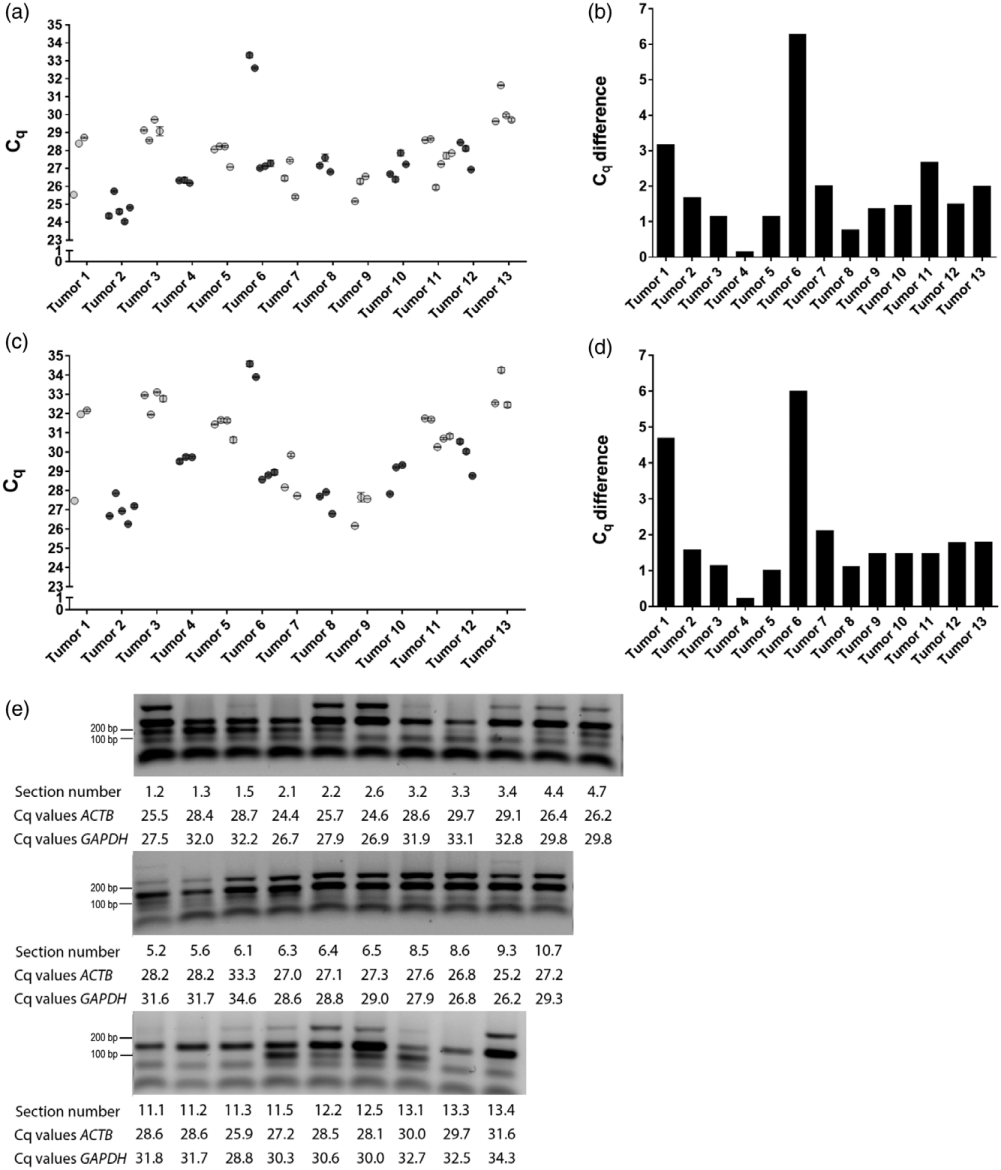
*ACTB* and *GAPDH* mRNA expression variation in sections from 13 primary melanoma tumors. (a and c) Display the raw inter-tumor Cq variation and (b and d) display the raw intra-tumor Cq variation of *ACTB* and *GAPDH*, respectively. (e) Displays variable length of *GAPDH* fragments amplified by RT-PCR. Base pair (bp) markers of 100 bps and 200 bps are shown at the left. Bottom panels showing corresponding Cq values for *ACTB* and *GAPDH* in qRT-PCR. Cq, quantification cycle.

Across the 13 melanomas investigated here, gene expression varied 9.27 Cq values for *ACTB* and 8.41 Cq for *GAPDH* (ΔCq values). The inter-tumor variation is displayed in Fig. 1a and c.

Intra-tumor variation analysis was based on a minimum of three sections sampled with a two-section interval. The expression of *ACTB* varied between 0.16 Cq and 6.29 Cq, and *GAPDH* expression varied between 0.23 and 6.01 Cq within tumors. The intra-tumor variation is displayed in Fig. 1b and d.

RNA quality and the degree of RNA degradation in each sample was assessed by multiplex RT-PCR amplification of *GAPDH* fragments of variable length. Results from *GAPDH* multiplex RT-PCR reactions for selected samples are shown in Fig. 1e. For tumors 1, 4, 5, 9, 10 and 12, the mRNA expression (Cq values measured by qRT-PCR) in all sections corresponded with the RNA quality assessed by *GAPDH* multiplex RT-PCR, where an increase in Cq value (decrease in expression) correlated with less amplification of the longer *GAPDH* fragments and vice versa. Intra-tumor *GAPDH*-specific Cq variation detected for these tumors may thus be caused by differences in RNA quality. This association between *GAPDH* mRNA expression and RNA quality was not observed for tumors 2, 3, 6, 8, 11 and 13. In some of the tumors with apparent high *GAPDH* mRNA expression as measured by qRT-PCR the RNA was actually vastly degraded. For example; in section 2.1, we measured high GAPDH mRNA expression (low Cq) by qRT-PCR compared to section 2.2 and 2.6, but the RNA quality for section 2.1 was poor compared to section 2.2 and 2.6. In section 3.4, we measured low *GAPDH* mRNA expression by qRT-PCR despite high quality of RNA measured by multiplex analysis. In section 11.3, we measured the highest expression of *GAPDH* mRNA (lowest Cq) by qRT-PCR; however the largest *GAPDH* fragment was not amplified from this section in multiplex RT-PCR indicating low RNA quality. Section 13.4 was the only section from tumor 13, from which we could amplify the largest *GAPDH* fragment; indicating higher RNA quality, however, amongst all the sections of tumor 13 the highest Cq (lowest *GAPDH* mRNA expression) was measured for section 13.4. Accordingly, we found no systematic association between the variation in gene expression levels (Cq values obtained by qRT-PCR) and the RNA quality (in terms of how long *GAPDH* fragments we could amplify in our multiplex RT-PCR).

**Fig. 2:**
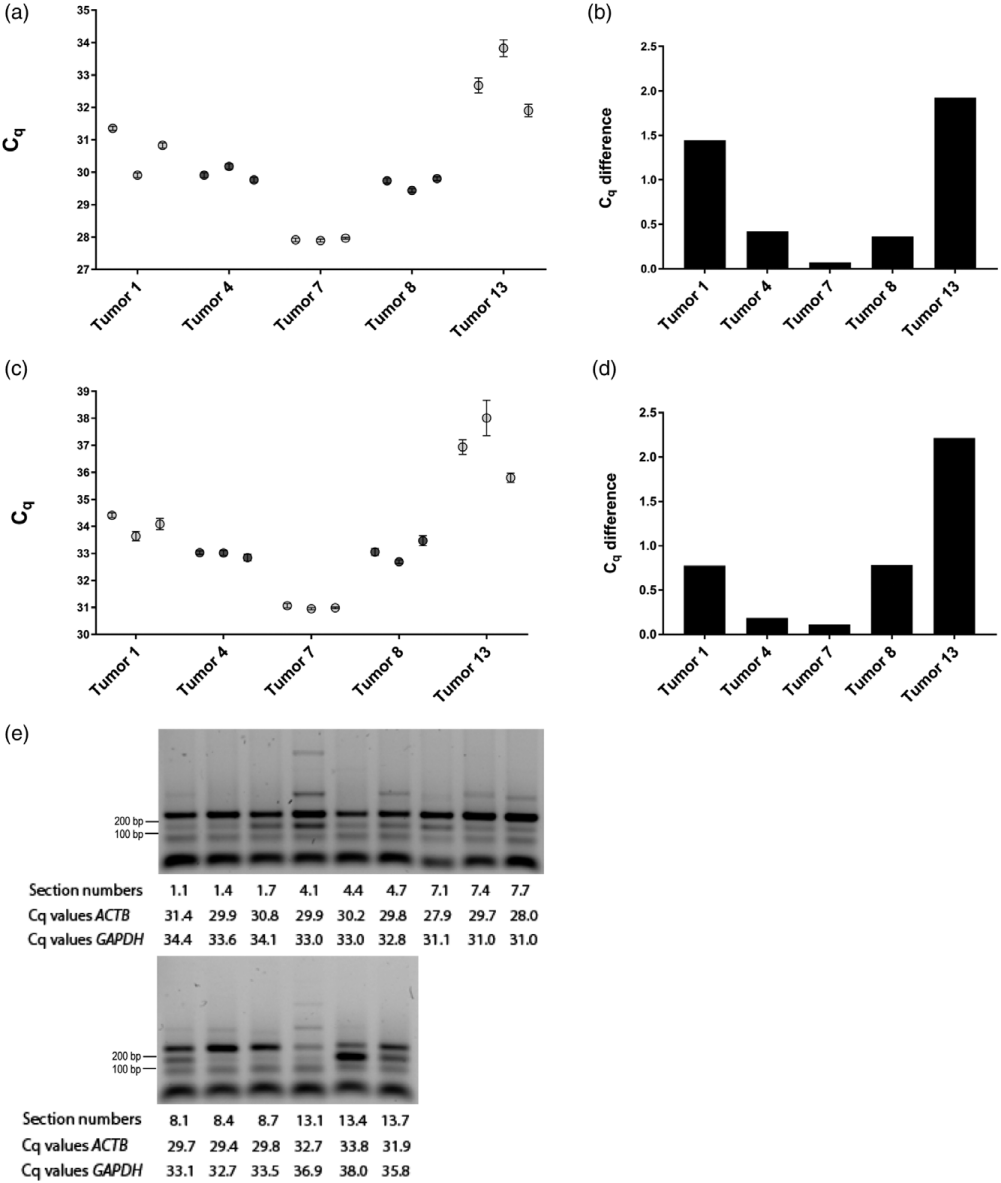
*ACTB* and *GAPDH* expression variation in macro-dissected non-cancerous epidermal tissue samples. (a and c) Display the raw inter-tumor Cq variation and (b and d) display the raw intra-tumor Cq variation of *ACTB* and *GAPDH*, respectively. The epidermal sections were prepared from patient samples of a selected subgroup of patients in the cohort presented in Fig. 1 (corresponding numbers between melanomas/patients and cutaneous sections are shown below each diagram). (e) Displays variable length of *GAPDH* fragments detected by RT-PCR. Bp markers are shown at the left. Bottom panels show corresponding Cq values for *ACTB* and *GAPDH*. The epidermal sections are from a selected group of patients from Fig. 1 (corresponding numbers between tumors and cutaneous sections are shown). Cq, quantification cycle.

To account for the contribution from cellular variations in the skin, for example, distribution of dermal cells and immunological infiltrates surrounding the tumor, to the variation in *ACTB* and *GAPDH* gene expression, we conducted RNA quality assessment by *GAPDH* multiplex RT-PCR and reference gene expression stability analyses by qRT-PCR using sections of non-cancerous cutaneous samples removed from five of the melanoma patients (1, 4, 7, 8 and 13). The measured expression of *ATCB* and *GAPDH* in the cutaneous samples varied up to 3.46 Cq and 3.47 Cq between samples, respectively (Fig. 2a and c). The intra-sample expression variation was between 0.07 and 1.92 Cq for *ACTB* and between 0.11 and 2.21 Cq for *GAPDH* (Fig. 2b and d). RNA isolated from the cutaneous samples from patients 1, 7 and 8 was of good quality and the reference gene expression corresponded well with the RNA quality (Fig. 2a, b and e). However, in section 4.1, higher mRNA quality was observed than for sections 4.4 and 4.7, but the GAPDH gene expression measured by qRT-PCR for sections 4.1, 4.4 and 4.7 was similar. Also, the *GAPDH* multiplex RT-PCR indicated low RNA quality in section 13.1, but qRT-PCR expression analysis resulted in Cq values of 32.7 for *ACTB* and 36.9 for *GAPDH*, which were substantially lower than for the corresponding section 13.4. It should also be noted that in general the average expression of *ACTB* and *GAPDH* the skin samples is fairly low compared to their expression in the melanoma samples. Accordingly, the contribution from the expression of these genes in normal skin is low compared to their expression in cancerous areas.

### Identification of robust reference genes suitable for studies of gene expression in FFPE primary melanomas

Even though we macrodissected all skin and melanoma sections before RNA extraction and only included RNA samples with comparable RIN values, we observed a large degree of variation in gene expression levels of *ACTB* and *GAPDH* across the 13 melanoma samples investigated. We did not find any systematic association between the variation in gene expression and RNA quality. This disqualified the genes as stand-alone normalizers of gene expression in melanoma. According to Bustin *et al*. [5], normalization against a single reference gene is not acceptable unless it is invariantly expressed across all samples under investigation.[Bibr R5] We therefore initiated a study with the purpose of identifying a panel of the most robust and stably expressed reference genes in FFPE primary melanomas for use as normalizers of melanoma gene expression in qRT-PCR-based analyses.

**Fig. 3:**
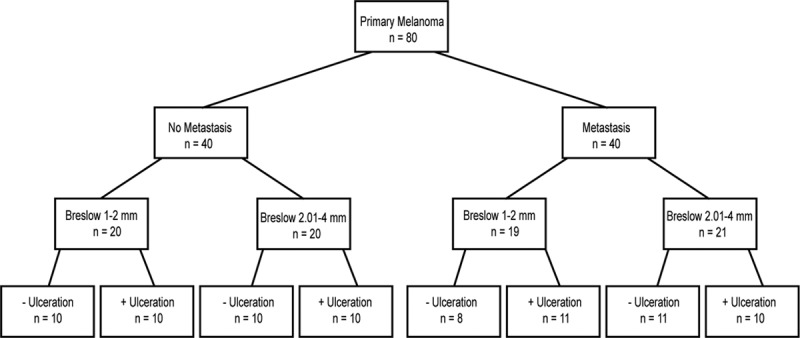
Overview of primary melanoma cohort used in the reference gene study. Inclusion criteria and numbers of included subjects in each subgroup.

We investigated the expression of these 24 candidate genes across 80 FFPE primary melanoma tumors selected across eight diagnostic subgroups. Figure 3 displays the grouping of patient samples included in this study. The qRT-PCR gene expression data were analyzed using geNorm and Normfinder algorithms to identify the most robust reference genes.

**Fig. 4:**
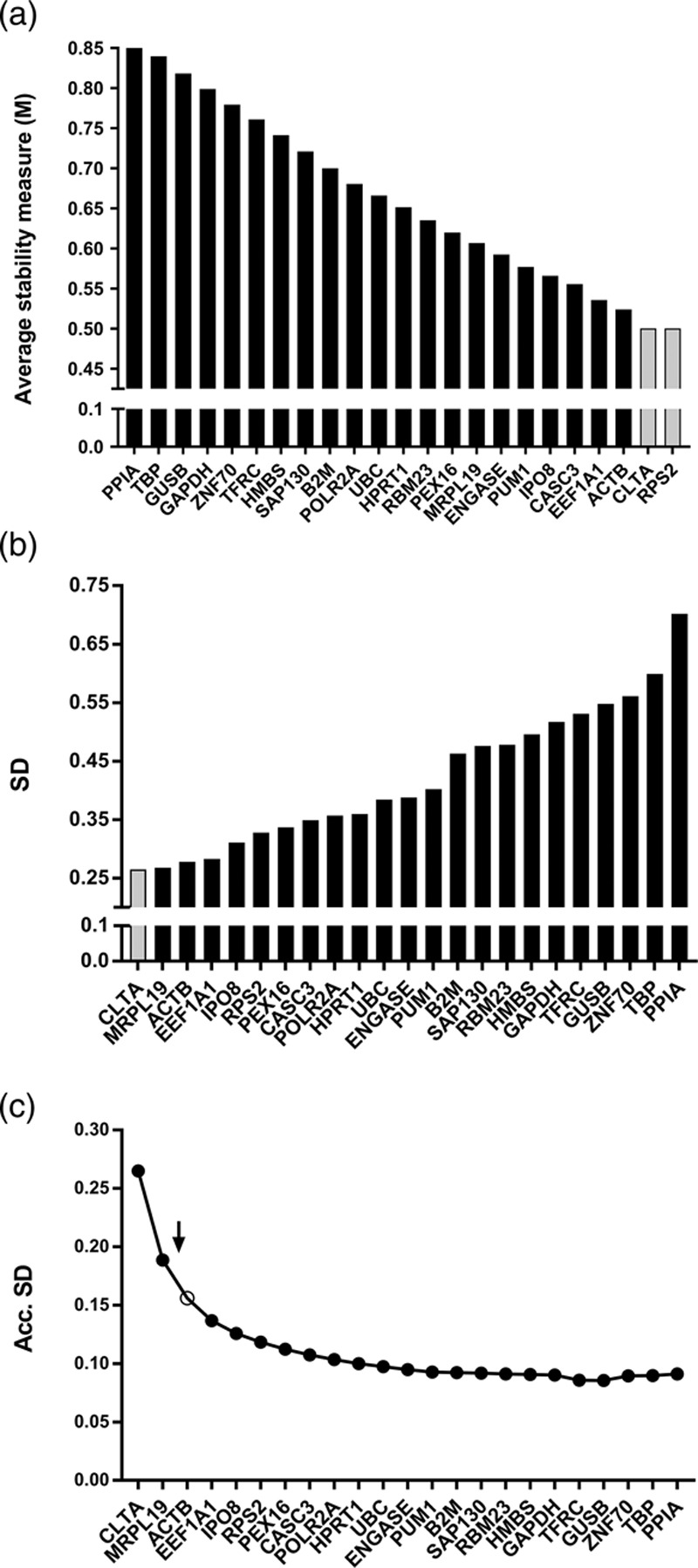
Evaluation of reference gene stability in melanoma tumors. (a) Displays the geNorm reference gene evaluation. The stability of the 24 candidate reference genes evaluated using the geNorm algorithm. The average stability measure M for the two most stable genes, *RPS2* and *CLTA*, is displayed in gray. The M values for the remaining genes are displayed in black. Gene names are indicated below the bars. In (b and c), the NormFinder reference gene evaluation is depicted. The stability of the 24 candidate reference genes evaluated using the NormFinder algorithm. In (b), the SD for the individual genes is shown across the 80 tumor samples without regard to subgroup. The most stable gene, *CLTA*, is displayed in gray, and the remaining genes are shown in black. Gene names are indicated below the bars. In (c) the stepwise inclusion of reference genes to the normalization factor is shown as a function of the accumulated SD indicating the improvement in stability. The inclusion of reference genes are marked by black dots and the black line indicates the accumulated SD.

The geNorm algorithm identified the two genes; *CLTA* and *RPS2*, as the most stable genes across the 80 tumors (n = 80) (average stability value M = 0.500) (Fig. 4a) (also see output in Table of Supplemental Digital Content 2, http://links.lww.com/MR/A184). Thus, the optimal NF according to geNorm is calculated as the geometric mean of the reference genes *CLTA* and *RPS2*.

The NormFinder algorithm identified *CLTA* and *MRPL19* as the optimal combination of reference genes when groups were taken into account (see data in Table of Supplemental Digital Content 3, http://links.lww.com/MR/A185). *CLTA* (stability value: 0.265) and *MRPL19* (stability value: 0.268) were also identified as the most optimal reference genes (accumulated SD = 0.189), when grouping was not accounted for (Fig. 4b). By use of NormFinder, the optimal number of reference genes to include in order to reduce the technical variation to an accepted level, can be determined based on calculated accumulated SDs by sequentially adding candidate reference genes to the normalizer. From Fig. 4c, it is evident that a substantial improvement of the *CLTA/MRPL19* normalizer can be achieved by the inclusion of *ACTB* as an additional reference gene (accumulated SD = 0.156) (also see data in Table of Supplemental Digital Content 4, http://links.lww.com/MR/A186). Additional reference genes may be added to the *CLTA/MRPL19/ACTB* NF; however from the sixth included reference gene, the additional decrease in technical variation per added gene is minimal.

### Identification of robust reference genes suitable for studies of gene expression in melanoma cell lines

We investigated the expression stability of *ACTB* and *GAPDH* across four melanoma cell lines; FM3, FM82, FM88 and FM92. *ACTB* and *GAPDH* expression (evaluated by ΔCq values ± standard error of the mean) varied 1.17 ± 0.05 and 1.21 ± 0.02 Cq, respectively (see output in Figure presented in Supplemental Digital Content 5, http://links.lww.com/MR/A189). The substantial variation in expression even when analyzing good quality starting material as fresh cell lysate enlightens that these two genes alone do not qualify as normalizers of target gene expression in qRT-PCR analyses in melanoma cells lines.

**Fig. 5 (Continued):**
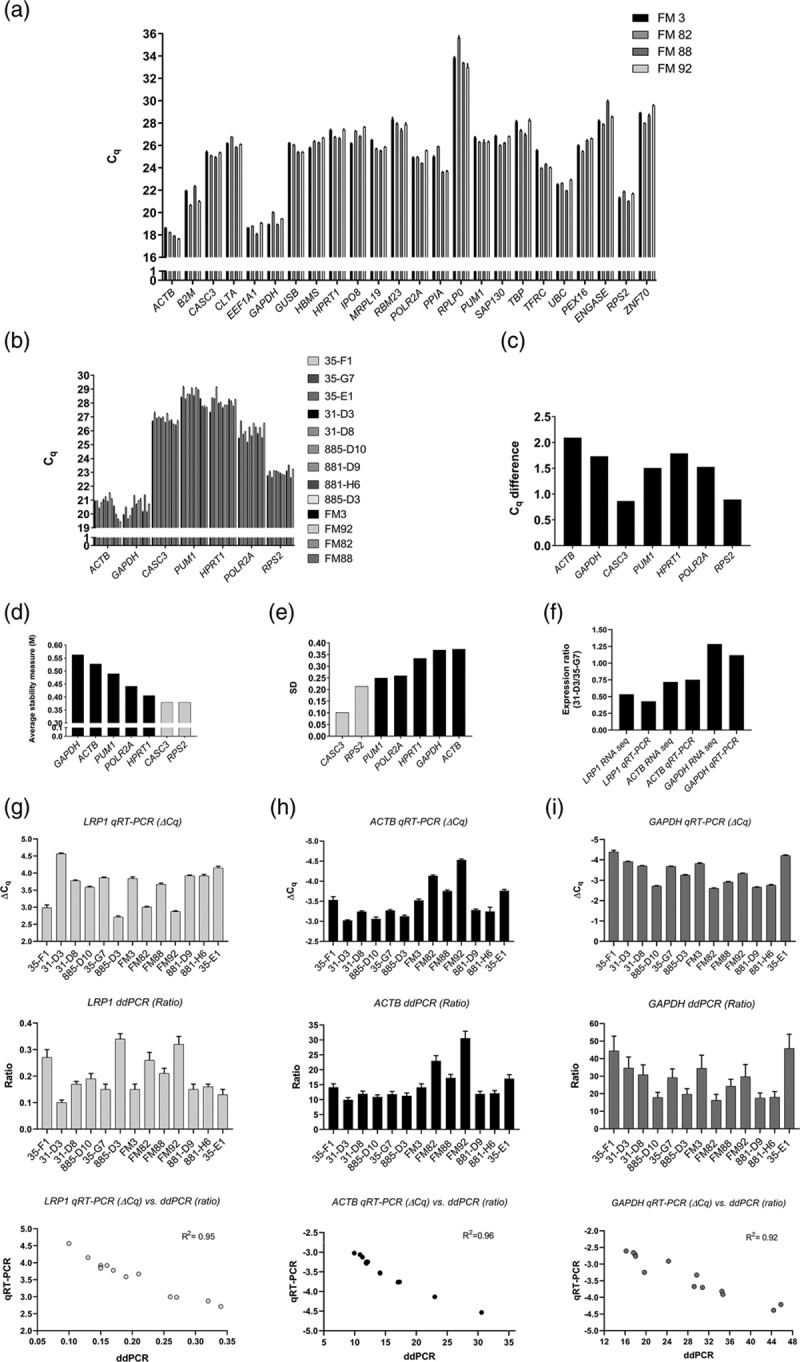
Evaluation of reference genes stability in melanoma cell lines. Expression variation of candidate reference genes in melanoma cell lines is displayed in (a–c). (a) Raw Cq values of the 24 candidate reference genes across the cultured melanoma cell lines; FM3, FM82, FM88 and FM92, measured by qRT-PCR. (b) Gene expression of the most stable genes from (a) across nine additional melanoma cell lines. (c) Differences in gene expression (ΔCq values) across all 13 melanoma cell lines. Cq, quantification cycle. For each gene, the total variation between measured Cq’s across all 13 melanoma cell lines is shown. In (d), the geNorm evaluation of reference genes is displayed. The seven candidate reference genes evaluated using the geNorm algorithm. The average stability measure M is displayed in gray for the two most stable genes, *RPS2* and *CASC3*, and in black for the remaining genes. Gene names are indicated below the bars. In (e), NormFinder evaluation of reference genes is displayed. Stability of the seven selected candidate reference genes across 13 melanoma cell lines without regard to genetic subgroup evaluated using the NormFinder algorithm. The most stable genes, *CASC3* and *RPS2*, are displayed in gray, and the remaining genes are shown in black. (f) RNAseq-based validation of qRT-PCR-based relative gene expression levels of *LRP1*, *ACTB* and *GAPDH* in 31-D3 and 35-G7 melanoma cells. qRT-PCR-based gene expression levels were normalized using reference genes *CASC3* and *RPS2*. RNAseq reads per gene were normalized to gene length. RNAseq expression ratios were calculated as the normalized gene read for each gene in 31-D3 divided by the normalized gene read for each gene in 35-G7. qRT-PCR expression ratios equals RQ values. (g) Upper panel: qRT-PCR ΔCqs for *LRP1* across 13 melanoma cell lines. Mid panel: ddPCR ratios for *LRP1* across 13 melanoma cell lines. Lower panel: correlation between qRT-PCR ΔCqs and ddPCR ratios. *P* < 0.0001. (h) Upper panel: qRT-PCR ΔCqs for *ACTB* across 13 melanoma cell lines. Mid panel: ddPCR ratios for *ACTB* across 13 melanoma cell lines. Lower panel: correlation between qRT-PCR ΔCqs and ddPCR ratios. *P* < 0.0001. (i) Upper panel: qRT-PCR ΔCqs for *GAPDH* across 13 melanoma cell lines. Mid panel: ddPCR ratios for *GAPDH* across 13 melanoma cell lines. Lower panel: correlation between qRT-PCR ΔCqs and ddPCR ratios. *P* < 0.0001. qRT-PCR- and ddPCR-based gene expression levels were normalized using a geometric mean of the expression of reference genes *CASC3* and *RPS2*. ddPCR, droplet digital-PCR; RNAseq, RNA sequencing; RQ, relative quantification.

We then tested the expression of each of the 24 candidate reference genes across the four melanoma cell lines, as shown in Fig. 5a. The five most stably expressed genes across the four melanoma cell lines were *CASC3* (0.51 ± 0.09), *PUM1* (0.44 ± 0.23), *HPRT1* (0.77 ± 0.10), *POLR2A* (1.14 ± 0.04) and *RPS2* (0.85 ± 0.04), as can be seen in Fig. 5b. We proceeded by analyzing the expression of the five aforementioned genes along with *ACTB* and *GAPDH* to enable a comparison of commonly used reference genes and carefully selected reference genes, across nine additional melanoma cell lines. The difference in expression of the five genes can be seen in Fig. 5c. The two most stably expressed genes were *CASC3* (0.87 ± 0.13) and *RPS2* (0.89 ± 0.03). The panel of genes was further evaluated using the geNorm and NormFinder algorithms to establish the strongest panel of reference genes for use as normalizers of melanoma cell line gene expression data.

The geNorm algorithm identified the two genes *CASC3* and *RPS2* as the most robust reference genes across the tested melanoma cell lines (n = 13) (average stability value M = 0.380) (output in Fig. 5d; also see data in Table of Supplemental Digital Content 6, http://links.lww.com/MR/A187). In melanoma cell lines, a geometric mean of the reference genes *CASC3* and *RPS2* is estimated to be the most optimal NF according to geNorm.

The NormFinder algorithm supported these result as *CASC3* (stability value: 0.102) and *RPS2* (stability value: 0.214) were identified to be the best and second-best reference gene, respectively, across the tested melanoma cell lines (n = 13) (Fig. 5e) (also see data in Table of Supplemental Digital Content 7, http://links.lww.com/MR/A188). Subsequent grouping of the parental and sub-cloned cell lines further resulted in identification of *CASC3* and *RPS2* as the best combination of reference genes (FM82, FM92, FM88 and sub-clones, n = 7; FM3 and sub-clones, n = 6).

Thus, based on the results from the two algorithms the most optimal NF for melanoma cell lines can be calculated as a geometric mean of reference genes *CASC3* and *RPS2*.

We compared our cell line results from the qRT-PCR-based analyses with corresponding data from other technological platforms, specifically RNAseq and ddPCR, and validated the reliability of *CASC3* and *RPS2* as reference genes. In Fig. 5f, the expression ratios obtained by RNAseq for target genes *LRP1*, *ACTB* and *GAPDH* in the cell line 31-D3 relative to 35-G7 was compared with the RQ values obtained by qRT-PCR-based measurements. By pairwise comparison of the results for each target gene, it is evident that we obtain a pattern of similar expression ratios across these two different technological platforms. In Fig. 5g, we compared the normalized expression levels for the target gene *LRP1* across 13 cell lines obtained by qRT-PCR-based measurements with the expression ratio obtained for the same target gene across the same 13 cell lines obtained by ddPCR. For both qRT-PCR- and ddPCR-based measurements, normalization of gene expression was conducted using *CASC3* and *RPS2* as reference genes. Very similar expression levels were obtained across the two different technological platforms, which is evident from an R^2^ of 0.95. In Fig. 5h and I, we compared the normalized expression level for the target genes *ACTB* and *GAPDH*, respectively, across 13 cell lines obtained by qRT-PCR-based measurements with the expression ratios obtained for the same target gene across the same 13 cell lines obtained by ddPCR. Again very similar relative expression levels were obtained across the two different technological platforms for both *ACTB* and *GAPDH* indicated by an R^2^ of 0.96 and 0.92, respectively.

*CASC3* and *RPS2* were also evaluated as reference genes in additional cancerous cell lines of melanocytic origin; WM115 and WM266-4, and in cell lines of non-melanocytic origin; HCC-70 (breast), and in BeWo, JEG-3 and JAR (of placental origin) (data not shown here), and even when these cell lines were included *CASC3* and *RPS2* were stably expressed across all samples investigated.

## Discussion

In this study, we have analyzed the intra- and inter-tumor expression stability of the two commonly used reference genes, *ACTB* and *GAPDH*, in 13 FFPE melanoma samples by qRT-PCR. We show that the two genes are not stably expressed across the 13 melanomas investigated here and not even within individual melanoma tumors. The observed intra-and inter-tumor differences in expression of the two genes were not caused by dissimilar RNA integrities of samples compared but rather by a genuine fluctuation in expression levels.

We also examined the expression of *ACTB* and *GAPDH* in non-cancerous epidermal tissue samples taken from the melanoma patients, and we found that the expression of *ATCB* and *GAPDH* vary substantially between the normal epidermal tissue samples as well. This demonstrates that even for gene expression analyses in non-cancerous homogeneous skin samples, *ACTB* and *GAPDH* alone do not qualify as stand-alone normalizers of gene expression.

We thus investigated the expression stability of 24 candidate reference genes in FFPE tissue samples collected from 80 melanoma patients. Patient samples from different diagnostic subgroups, counting tumors that were either BRAF mutated or not, tumors with or without ulceration, and thin as well as thick tumors were included. We applied two different mathematical algorithms for evaluation and comparison of the candidate reference genes. We identified *CLTA* as the most robust reference gene for normalization of gene expression in primary melanomas followed by *MRPL19* and *ACTB*, respectively.

However, the use of only a single reference gene for normalization of target gene expression data is not recommendable, especially not when analyzing tissue biopsies [37]. Instead, a multigene NF should be used, which is achieved by combining the expression of a number of validated genes [38]. The use of a multigene NF reduces the impact of fluctuations in a single reference gene resulting in higher quality results [38]. Based on our data, we recommend the use of a combined geometric mean of the expression levels of *CLTA*, *MRPL19* and *ACTB* for normalization of gene expression in FFPE melanomas. Inclusion of additional reference genes to this combined NF might improve it slightly, but as indicated in Fig. 4c, it will not decrease the variation substantially. Thus, with cost-benefits in mind we recommend a NF including these three genes (*CLTA*, *MRPL19* and *ACTB*) only. To our knowledge, our study is the first to systematically identify and validate a panel of robust reference genes for normalization of gene expression in primary melanomas.

For studies of gene expression in melanoma cell lines; we identified *CASC3* and *RPS2* as robust reference genes over 13 different melanoma cells lines. We recommend the use of a combined geometric mean of the expression levels of *CASC3* and *RPS2* for normalization of gene expression in melanoma cell lines.

We further compared gene expression data from different technical platforms. By comparing the relative expression ratios of target genes *LRP1*, *ACTB* and *GAPDH* across two melanoma cell lines estimated using an RNAseq approach and a qRT-PCR-based approach, we show that indeed we obtain similar relative gene expression levels of the target genes. We further compared the relative gene expression ratios of target genes *LRP1*, *ACTB* and *GAPDH* across 13 melanoma cell lines estimated by qRT-PCR or ddPCR. We find a strong correlation between data from qRT-PCR and ddPCR for each target gene demonstrating robustness of the reference genes *CASC3* and *RPS2* across technological platforms.

*CASC3* and *RPS2* also were evaluated as reference genes in yet two other melanoma cell lines and a number of other cell lines of origin different from melanoma (breast and placenta), and they were found to be robustly expressed in all cases. These two genes might therefore also qualify as normalizers of gene expression in cell lines of origin different from melanoma.

The discrepancy between the optimal reference genes identified for normalization of gene expression in FFPE melanomas and cultured melanoma cells and the different number of reference genes required to be included in the NF might be explained in two ways. First is the obvious difference in the quality of starting material. The integrity of the RNA is normally very high in fresh-frozen cell lysates, whereas the RNA integrity of FFPE tissue specimens is often very low [39,40]. Next, each investigated melanoma cell line is relatively homogenous, whereas melanoma tumors are highly heterogeneous entities [41–50].

In conclusion, this study has now established a panel of robust reference genes for use as normalizers in melanoma gene expression studies. This will hopefully pave the way for trustworthy, efficient and successful analyses of gene expression in primary melanomas regardless of diagnostic subgroup and pave the way for validation of novel and better melanoma biomarkers.

## Acknowledgements

We thank Dr. Marie Louise Bonnelykke Behrndtz, Aarhus University Hospital for assistance in selection of melanoma samples and Søren Egedal Degn and Thomas Wittenborn for assistance on ddPCR matters.

This study was supported by grants from Emil C. Hertz-, Georg Bjørkner-, Holm-, Einar Willumsen’s-, Frænkels- and Wedell-Erichsen’s foundations.

## Conflicts of interest

There are no conflicts of interest.

## Supplementary Material
