# Supplementary material for: Identification of robust reference genes for studies of gene expression in FFPE melanoma samples and melanoma cell lines
Source: Melanoma Res. 2019 Sep 24;30(1):26–38. doi: 10.1097/CMR.0000000000000644 (PMC6940030; doi:10.1097/CMR.0000000000000644)
Supplement: Supplementary file 1 [file mr-30-26-s001.pdf]

Supplemental digital content 2

geNorm gene ranking		
Rank	Gene name	M-value
1	RPS2	0,500
2	CLTA	0,500
3	ACTB	0,524
4	EEF1A1	0,536
5	CASC3	0,556
6	IPO8	0,566
7	PUM1	0,577
8	ENGASE	0,593
9	MRPL19	0,607
10	PEX16	0,620
11	RBM23	0,635
12	HPRT1	0,652
13	UBC	0,666
14	POLR2A	0,681
15	B2M	0,700
16	SAP130	0,721
17	HMBS	0,741
18	TFRC	0,761
19	ZNF70	0,779
20	GAPDH	0,799
21	GUSB	0,818
22	TBP	0,840
23	PPIA	0,869
