# Supplementary material for: Identification of robust reference genes for studies of gene expression in FFPE melanoma samples and melanoma cell lines
Source: Melanoma Res. 2019 Sep 24;30(1):26–38. doi: 10.1097/CMR.0000000000000644 (PMC6940030; doi:10.1097/CMR.0000000000000644)
Supplement: Supplementary file 2 [file mr-30-26-s002.pdf]

Supplemental digital content 3

A. NormFinder intragroup expressional variation								
**Group identifier**	1	2	3	4	5	6	7	8
**Gene**								
ACTB	0,023	0,111	0,082	0,020	0,040	0,038	0,107	0,096
B2M	0,310	0,058	0,466	0,147	0,185	0,104	0,396	0,072
CASC3	0,071	0,030	0,294	0,034	0,078	0,143	0,019	0,288
CLTA	0,067	0,016	0,109	0,075	0,023	0,027	0,074	0,036
EEF1A1	0,148	0,048	0,071	0,070	0,029	0,005	0,153	0,062
GAPDH	0,276	0,200	0,345	0,291	0,195	0,245	0,250	0,356
GUSB	0,170	0,183	0,951	0,144	0,382	0,256	0,112	0,280
HMBS	0,164	0,071	0,304	0,200	0,173	0,254	0,356	0,161
HPRT1	0,053	0,065	0,104	0,227	0,147	0,183	0,034	0,110
IPO8	0,101	0,064	0,207	0,096	0,035	0,065	0,093	0,099
MRPL19	0,067	0,043	0,031	0,118	0,058	0,039	0,025	0,147
RBM23	0,194	0,265	0,190	0,355	0,116	0,154	0,076	0,194
POLR2A	0,120	0,061	0,267	0,184	0,018	0,105	0,086	0,042
PPIA	0,130	0,388	1,671	0,177	0,923	0,445	0,433	0,239
PUM1	0,106	0,061	0,190	0,176	0,135	0,235	0,122	0,138
SAP130	0,118	0,150	0,099	0,130	0,196	0,274	0,442	0,456
TBP	0,279	0,171	0,108	0,491	0,912	0,047	0,829	0,255
TFRC	0,226	0,119	0,196	0,304	0,110	0,238	0,656	0,234
UBC	0,034	0,070	0,184	0,184	0,046	0,196	0,098	0,287
PEX16	0,104	0,043	0,034	0,187	0,051	0,101	0,282	0,138
ENGASE	0,108	0,145	0,265	0,246	0,071	0,162	0,142	0,109
RPS2	0,094	0,125	0,158	0,062	0,122	0,063	0,172	0,033
ZNF70	0,134	0,102	0,105	0,091	0,362	0,416	0,045	0,984

B. NormFinder intergroup expressional variation								
**Group identifier**	1	2	3	4	5	6	7	8
**Gene**								
ACTB	-0,021	0,179	0,163	-0,128	0,047	0,011	-0,223	-0,027
B2M	0,110	-0,065	0,017	-0,092	0,283	-0,056	-0,142	-0,054
CASC3	0,019	0,033	0,184	0,073	-0,036	-0,156	0,056	-0,173
CLTA	0,181	-0,185	0,053	-0,246	-0,046	0,077	0,132	0,035
EEF1A1	0,165	-0,093	0,153	-0,084	0,066	-0,119	-0,006	-0,082
GAPDH	0,054	-0,125	-0,207	0,027	-0,112	0,071	0,019	0,274
GUSB	-0,135	0,083	-0,086	-0,059	-0,129	-0,042	0,025	0,344
HMBS	-0,184	0,002	-0,021	-0,047	-0,389	0,396	0,020	0,223
HPRT1	-0,311	-0,010	-0,037	0,196	-0,042	-0,083	0,179	0,108
IPO8	-0,054	-0,084	-0,024	0,192	0,089	0,054	-0,048	-0,124
MRPL19	-0,161	0,052	0,005	0,251	-0,029	-0,070	-0,039	-0,008
RBM23	0,034	0,292	0,152	0,071	0,087	-0,209	0,035	-0,463
POLR2A	0,071	-0,013	-0,273	-0,203	0,041	0,082	0,062	0,233
PPIA	0,080	0,028	-0,118	0,010	-0,058	0,044	0,016	-0,003
PUM1	0,020	0,073	0,157	0,170	0,122	-0,068	-0,147	-0,327
SAP130	0,227	0,042	-0,109	-0,065	-0,128	0,021	-0,009	0,022
TBP	0,071	0,005	-0,241	0,015	-0,100	0,062	-0,059	0,248
TFRC	0,087	-0,049	-0,442	-0,062	0,105	0,073	0,168	0,119
UBC	0,032	-0,091	-0,245	0,005	0,099	0,221	-0,107	0,088
PEX16	-0,027	0,099	0,100	0,011	-0,058	-0,025	-0,069	-0,031
ENGASE	-0,007	-0,005	0,045	0,131	0,011	-0,126	0,059	-0,107
RPS2	-0,083	-0,019	0,260	-0,085	-0,051	-0,113	0,072	0,019
ZNF70	-0,166	-0,149	0,515	-0,081	0,230	-0,045	0,006	-0,311
