# Supplementary material for: Identification of robust reference genes for studies of gene expression in FFPE melanoma samples and melanoma cell lines
Source: Melanoma Res. 2019 Sep 24;30(1):26–38. doi: 10.1097/CMR.0000000000000644 (PMC6940030; doi:10.1097/CMR.0000000000000644)
Supplement: Supplementary file 3 [file mr-30-26-s003.pdf]

Supplemental digital content 4

NormFinder gene ranking			
Rank	Gene name	Stability value	Accumulated SD
1	CLTA	0,265	0,265
2	MRPL19	0,268	0,189
3	ACTB	0,278	0,156
4	EEF1A1	0,283	0,137
5	IPO8	0,311	0,126
6	RPS2	0,328	0,118
7	PEX16	0,337	0,112
8	CASC3	0,349	0,108
9	POLR2A	0,357	0,103
10	HPRT1	0,360	0,100
11	UBC	0,384	0,097
12	ENGASE	0,388	0,095
13	PUM1	0,402	0,093
14	B2M	0,463	0,092
15	SAP130	0,476	0,092
16	RBM23	0,478	0,091
17	HMBS	0,496	0,091
18	GAPDH	0,517	0,090
19	TFRC	0,531	0,086
20	GUSB	0,548	0,085
21	ZNF70	0,561	0,090
22	TBP	0,599	0,090
23	PPIA	0,702	0,091
