# Supplementary material for: Identification of robust reference genes for studies of gene expression in FFPE melanoma samples and melanoma cell lines
Source: Melanoma Res. 2019 Sep 24;30(1):26–38. doi: 10.1097/CMR.0000000000000644 (PMC6940030; doi:10.1097/CMR.0000000000000644)
Supplement: Supplementary file 4 [file mr-30-26-s004.pdf]

Supplemental digital content 6

geNorm gene ranking		
**Rank**	**Gene name**	**M-value**
1	RPS2	0,380
2	CASC3	0,380
3	HPRT1	0,406
4	POLR2A	0,442
5	PUM1	0,490
6	ACTB	0,528
7	GAPDH	0,563
