# Supplementary material for: Identification of robust reference genes for studies of gene expression in FFPE melanoma samples and melanoma cell lines
Source: Melanoma Res. 2019 Sep 24;30(1):26–38. doi: 10.1097/CMR.0000000000000644 (PMC6940030; doi:10.1097/CMR.0000000000000644)
Supplement: Supplementary file 5 [file mr-30-26-s005.pdf]

Supplemental digital content 7

NormFinder gene ranking			
**Rank**	**Gene name**	**Stability value**	**Accumulated SD**
1	CASC3	0,102	0,102
2	RPS2	0,214	0,118
3	PUM1	0,250	0,115
4	POLR2A	0,260	0,108
5	HPRT1	0,334	0,109
6	GAPDH	0,370	0,110
7	ACTB	0,374	0,108
