# Supplementary material for: Identification of robust reference genes for studies of gene expression in FFPE melanoma samples and melanoma cell lines
Source: Melanoma Res. 2019 Sep 24;30(1):26–38. doi: 10.1097/CMR.0000000000000644 (PMC6940030; doi:10.1097/CMR.0000000000000644)
Supplement: Supplementary file 7 [file mr-30-26-s007.pdf]

Supplemental digital content 1

Sample Name	RIN	Sample Name	RIN	Sample Name	RIN
Melanoma samples		*Melanoma samples*		*Melanoma samples*	
1	1.40	33	2.40	65	2.20
2	1.00	34	1.80	66	2.50
3	1.00	35	2.30	67	1.00
4	1.50	36	1.20	68	1.60
5	1.20	37	2.40	69	1.10
6	1.20	38	2.40	70	2.10
7	2.50	39	2.40	71	2.40
8	1.60	40	2.00	72	2.40
9	1.40	41	1.10	73	2.50
10	2.30	42	1.10	74	2.40
11	2.30	43	1.10	75	2.50
12	2.40	44	1.30	76	1.80
13	2.00	45	1.80	77	2.40
14	2.50	46	2.40	78	2.40
15	1.30	47	2.20	79	2.30
16	1.80	48	2.00	80	2.10
17	1.10	49	2.30	*Cell lines*	
18	2.50	50	1.70	35-F1	10.00
19	2.10	51	2.20	885-D3	9.70
20	2.40	52	1.00	885-D10	10.00
21	2.40	53	2.00	31-D8	10.00
22	2.50	54	2.20	881-H6	10.00
23	2.00	55	1.30	881-D9	10.00
24	1.80	56	2.40	FM3	9.90
25	2.40	57	2.20	FM92	10.00
26	1.90	58	1.60	FM82	10.00
27	2.50	59	2.50	31-D3	10.00
28	1.80	60	2.00	FM88	10.00
29	2.00	61	1.80	35-E1	10.00
30	2.10	62	1.90	35-G7	10.00
31	2.30	63	2.40		
32	2.40	64	2.00		
